# Predictive Value of the Rapid Emergency Medicine Score (REMS) for Non-trauma Geriatric Patients Presenting to the Emergency Department: A Prospective Observational Study

**DOI:** 10.7759/cureus.83853

**Published:** 2025-05-10

**Authors:** Puneeth M Reddy, Karthik Reddy CH, Brian William Dmello, Mary Jacob, Girish Narayan, Shakuntala Murty K

**Affiliations:** 1 Emergency Medicine, Rohilkhand Medical College and Hospital, Bareilly, IND; 2 Emergency Medicine, St. John's Medical College, Bangalore, IND; 3 Emergency Medicine, The Oxford Medical College, Hospital & Research Centre, Bangalore, IND; 4 Emergency Medicine, Amala Institute of Medical Sciences, Thrissur, IND

**Keywords:** emergency medical service, geriatric patients, morbidity, mortality, rapid emergency medicine score (rems)

## Abstract

Background: In the future, countries such as India are expected to experience an increase in the number of elderly patients with medical conditions in the emergency department (ED) due to demographic changes. Increased mortality, institutionalization, hospital readmission, and functional impairment are some of the potentially severe outcomes associated with hospitalization for some of these older patients. Through this study, we aimed to evaluate the utility of the Rapid Emergency Medicine Score (REMS) in predicting morbidity and in-hospital mortality among geriatric patients presenting to the ED.

Methods: A prospective observational study was conducted in the Department of Emergency Medicine from November 2020 to March 2022. The data were retrieved from the records of patients who presented to the ED. After obtaining the necessary clearance from the hospital's ethical review committee, follow-up data were subsequently collected from the in-patient records of these patients.

Results: REMS demonstrated a sensitivity of 50.57% and a specificity of 77.88% for predicting intensive care unit (ICU) admissions. The calculated area under the curve (AUC) for REMS in this study was 0.68, with a confidence interval of 0.60-0.75. We also compared the AUC of both REMS and modified Rapid Emergency Medicine Score (mREMS), and no difference was found between the two scores. As per the results, the REMS score predicted morbidity and mortality in geriatric patients presenting to the ED with low sensitivity and equivocal specificity.

Conclusion: REMS is a relatively reliable predictor of the likelihood of admission, particularly for ICU admissions, and a moderately effective predictor for admitting patients to the ward with a sensitivity of 50%.

## Introduction

In young patients admitted to the emergency department (ED), clinical factors directly associated with the acute illness are typically decisive in determining the outcome. In contrast, the prognosis for elderly individuals is less straightforward, as it is influenced by a range of factors that extend beyond the severity of the acute condition itself [1]. Specifically, these factors encompass functional, emotional, and cognitive states, the extent of comorbidities, the degree of polypharmacy, and the availability of social support networks [2]. Due to their inherent vulnerability and frailty, the health challenges faced by elderly patients are best understood through a multifaceted framework, wherein their resolution is not attributable to a single factor but rather to the comprehensive identification and management of all contributing elements that impact their prognosis [3]. In this context, implementing an objective scoring system would prove invaluable in accurately guiding the clinical decision-making process and determining the most appropriate disposition for such patients [4].

In the future, we can expect an increase in the number of elderly patients with medical conditions presenting to the ED due to demographic shifts [5,6]. Increased mortality, institutionalization, hospital readmission, and functional impairment are some of the potentially severe outcomes associated with hospitalization for some of these older patients [7]. In resource-constrained settings, such as India, where medical facilities are often limited, risk assessment in the ED becomes crucial [8,9]. This enables emergency physicians to make informed decisions regarding the need for intensive care unit (ICU) admission, thereby facilitating early and accurate counseling of families [10]. Given the significant financial implications associated with ICU care, the ability to predict the necessity for such intervention through a reliable scoring system would be invaluable. It would not only assist in optimizing resource allocation but also enable proactive communication with the patient's family, providing them with a clearer understanding of the situation and potential costs, thereby mitigating the financial burden and enhancing informed decision-making [11]. The Rapid Emergency Medicine Score (REMS) is a quick, easy-to-use scoring tool based on data readily available in the ED and is unaffected by future treatments. Scoring systems like the REMS have been widely used due to their readily available physiological parameters, including age, the Glasgow Coma Scale (GCS), respiratory rate, oxygen saturation, mean arterial pressure (MAP), and heart rate [12]. While systems like the Acute Physiology and Chronic Health Evaluation II (APACHE II) are widely used in ICUs for patient stratification, performance comparison, and resource analysis, REMS could serve similar roles in emergencies [12].

Through this study, we aim to predict the value of the REMS in geriatric patients (aged 65 years and above) presenting to the ED of a tertiary care hospital and to assess the risk of ICU admission in geriatric patients in the ED for effective family counseling. Furthermore, this study aimed to estimate the risk of ICU admissions, morbidity, and in-hospital mortality in geriatric patients presenting to the ED using the REMS score.

## Materials and methods

A prospective observational study was undertaken at the ED of St. John's Medical College and Hospital, Bangalore, India, from November 2020 to March 2022. The institute's Institutional Ethics Committee approved the study (study reference number: 373/2020). Informed consent was obtained from all enrolled patients.

Although the World Health Organization defines geriatrics as individuals aged 65 and above, the threshold is often lower in the Indian context. To ensure generalizability and consistency with prior research, this study adopts the global standard of 65 years, advocating for a unified age definition in geriatric research and policy. Hence, patients aged 65 or older who presented to the ED during the study period with medical or surgical diagnoses were included. In contrast, trauma patients aged 65 years and above were excluded due to differing clinical profiles and the need for specialized scoring systems. Patients who had received or receiving intravenous fluids and/or inotropes or inotropic support for hemodynamic instability before presentation to the ED and those who were intubated and mechanically ventilated before ED arrival were also excluded, as these interventions could affect initial vital signs and compromise the accuracy of REMS scoring.

The data source was the ED records of patients, and further follow-up data was retrieved from the in-patient records of these patients. The clinical parameters, including age, pulse rate, respiratory rate, GCS score, blood pressure, and oxygen saturation, were recorded, and the REMS score was calculated for each patient at presentation (see Appendices). In calculating the REMS score, a baseline score of 5 was assigned to all patients, reflecting the study population's age being greater than 65 years. For patients aged above 74 years, a score of 6 was applied in place of 5, in accordance with the REMS age-based scoring criteria.

Statistical analysis

Dundar et al. demonstrated that the REMS scoring method for identifying the need for ICU admission in elderly patients has a sensitivity and specificity of 70%. To achieve a similar sensitivity of 70% with 10% precision and a 95% confidence interval (CI), a sample size of 200 patients was required [13]. It was assumed that approximately 30% of the patient population under assessment would require ICU admission [4]. Based on this, a minimum of 200 patients were included in the study.

Data were entered into a single Microsoft Excel spreadsheet (Microsoft Corp., Redmond, WA, USA), and the sensitivity and specificity of the REMS score were calculated. The ability of the REMS score to predict ICU admission and in-hospital mortality was evaluated using receiver operating characteristic (ROC) curve analysis. Additionally, the sensitivity, specificity, and ROC curve values for the REMS score were calculated using IBM SPSS Statistics for Windows, V. 20.0 (IBM Corp., Armonk, NY, USA). A p-value of 0.05 was considered the threshold for statistical significance in all tests.

## Results

Study population and baseline characteristics

A total of 200 patients were recruited during the study period. Simple random sampling was employed, and the recruitment of the patients was stopped when the sample size reached 200.

Of these, the number of male patients was 120 (60%), and the mean age of the dataset was 72.12 years, with a standard deviation of 6.14 years, as illustrated in Table 1.

**Table 1:** Characteristics of the study population and their distribution. MAP: mean arterial pressure; GCS: Glasgow Coma Scale

Characteristics	Mean values	Standard deviation
Age	72.12	6.14
Pulse rate	89.08	20.7
Respiratory rate	21.18	5.06
Systolic blood pressure	136.7	28.11
Diastolic blood pressure	85.31	15.7
MAP	102.44	18.3
Saturation	92.18	5.72
GCS	14.33	1.79

Distribution of REMS score components

Table 2 provides a comprehensive overview of the different components that make up the REMS score. It outlines each variable included in the score, such as pulse rate, MAP, respiratory rate, and GCS, along with the corresponding scoring system.

**Table 2:** Components of the Rapid Emergency Medicine Score. PR: pulse rate; RR: respiratory rate; MAP: mean arterial pressure; GCS: Glasgow Coma Scale; SpO2: peripheral oxygen saturation

Variable/score	0	+1	+2	+3	+4	+5	+6
Age (years)	<45	-	45-54	55-64	-	65-74	>74
PR (/min)	70-109	-	55-69 or 110-139	40-54 or 140-179	<39 or >179	-	-
MAP (mmHg)	70-109	-	50-69 or 110-129	130-159	<49 or >159	-	-
RR (/min)	12-24	10-11 or 25-34	6-9	35-49	<5 or >49	-	-
GCS	14 or 15	11-13	8-10	5-7	3 or 4	-	-
SpO2 (%)	>89	86-89	-	75-85	<75	-	-

The patients were given a score for each variable, and the total score was calculated. Most of the population was between 65 and 74 years of age, i.e., 147 (73.5%) and 26.5% (53), with an age above 74 years. When analyzing the individual components of the REMS score, 130 patients (65%) had a pulse rate within the range of 70-109 bpm. The mean pulse rate was 89.8 bpm, with a standard deviation of 20.7. The mean pulse rate was 89.8, with a standard deviation of 20.7. Most of the patients had a MAP between 70 and 109 mmHg (64.5%). One hundred and thirty-seven (68%) patients out of 200 fell under the score of 0 (12-24 bpm). One hundred and sixty-nine (84.5%) of the 200 patients had a GCS score of 14 or 15 (score 0). About 72.5% of the subjects had a REMS score of 0, 15.5% with a REMS score of 1, 11% with a REMS score of 3, and one person with a REMS score of 4 for the variable of saturation.

Comorbidities in the study population

The number of patients with cerebrovascular accident (CVA) was 159 (79.5%) when compared to 41 (20.5%) who did not have CVA. The number of patients with hypertension was 150 (76%) when compared to patients without hypertension, who were 48 (34%). The number of patients having diabetes mellitus (DM) when compared to patients not having DM was 110 (55%) and 90 (45%), respectively.

Odds ratios for ICU admission

Table 3 presents the crude odds ratio and the odds ratio adjusted for all comorbidities and sex. All odds ratios are more than 1, and all p-values are significant. A base score of 5 was added to each patient's REMS score since the study population was over 65 years of age. For patients older than 74, a score of 6 was added instead.

**Table 3:** OR adjusted for comorbidities and sex. OR: odds ratio; HTN: hypertension; CVA: cerebrovascular accident; DM: diabetes mellitus

Variables	OR	Confidence interval	P-value
Crude OR	3.60	1.95-6.63	0.00
OR adjusted for sex	3.55	1.92-6.57	0.00
OR adjusted for HTN	3.55	1.91-6.57	0.00
OR adjusted for CVA	3.60	1.95-6.64	0.00
OR adjusted for DM	3.69	1.99-6.83	0.00

ICU admissions based on REMS score

Table 4 presents the 2 × 2 table of REMS scores, categorized by scores of 8 or less and those greater than 8, along with the number of patients admitted to the ward and ICU. Among the 25 patients admitted to the ward with a REMS score greater than 8, 17 (68%) were transferred to the ICU within a few days of admission.

**Table 4:** REMS score and disposition. ICU: intensive care unit; REMS: Rapid Emergency Medicine Score

Disposition (REMS score)	Ward	ICU
=8	88	43
>8	25	44

Statistical analysis

In the primary analysis, the REMS score used to detect the morbidity (ICU admissions) of the patients with age >65 had a sensitivity of 50.57% (95% CI: 43.65-57.5%) and a specificity of 77.88% (95% CI: 72.12-83.63%) (Table 5).

**Table 5:** Statistical analysis of the REMS score of this study. CI: confidence interval; REMS: Rapid Emergency Medicine Score

Statistics	Values	95% CI
Sensitivity	50.57%	43.65-57.5%
Specificity	77.88%	72.12-83.63%
Positive predictive value	63.77%	57.11-70.43%
Negative predictive value	67.18%	60.67-73.68%

The study also had a positive predictive value of 63.77% (95% CI: 57.11-70.43%) and a negative predictive value of 67.18% (95% CI: 60.67-73.68%).

Area under the curve (AUC) analysis of REMS and modified Rapid Emergency Medicine Score (mREMS)

**Figure 1:**
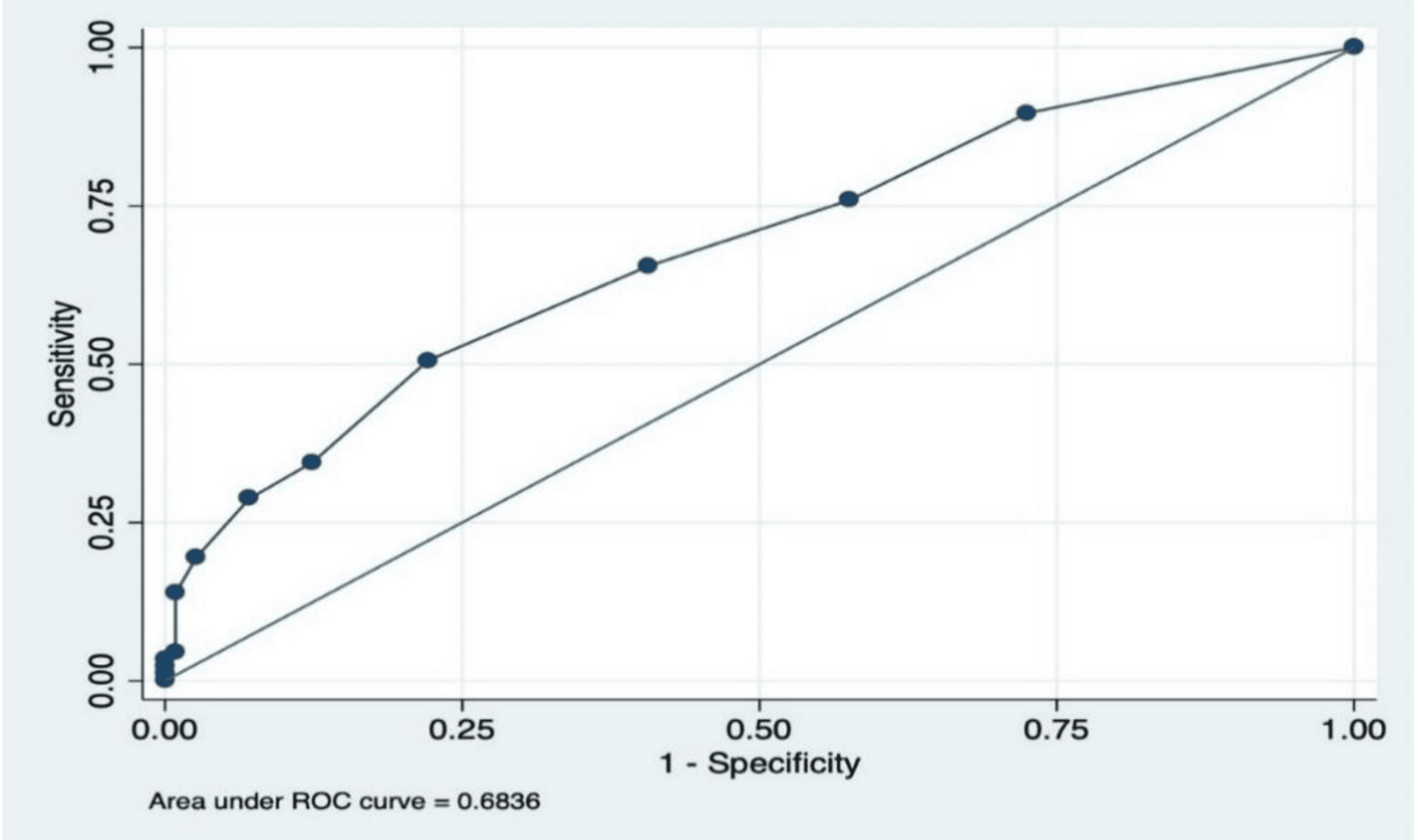
The AUC for REMS, which was 0.6836, with a confidence interval ranging from 0.60 to 0.75. This demonstrates that REMS has a moderate ability to predict the need for patient admission. AUC: area under the curve; REMS: Rapid Emergency Medicine Score; ROC: receiver operating characteristic

In this study, the AUC for the REMS was calculated to be 0.68 (Figure 1) with a CI ranging from 0.60 to 0.75. This AUC value was lower than reported in previous surveys, indicating a slightly reduced predictive performance in this dataset. Still, it has a moderate ability to predict the need for patient admission.

**Figure 2:**
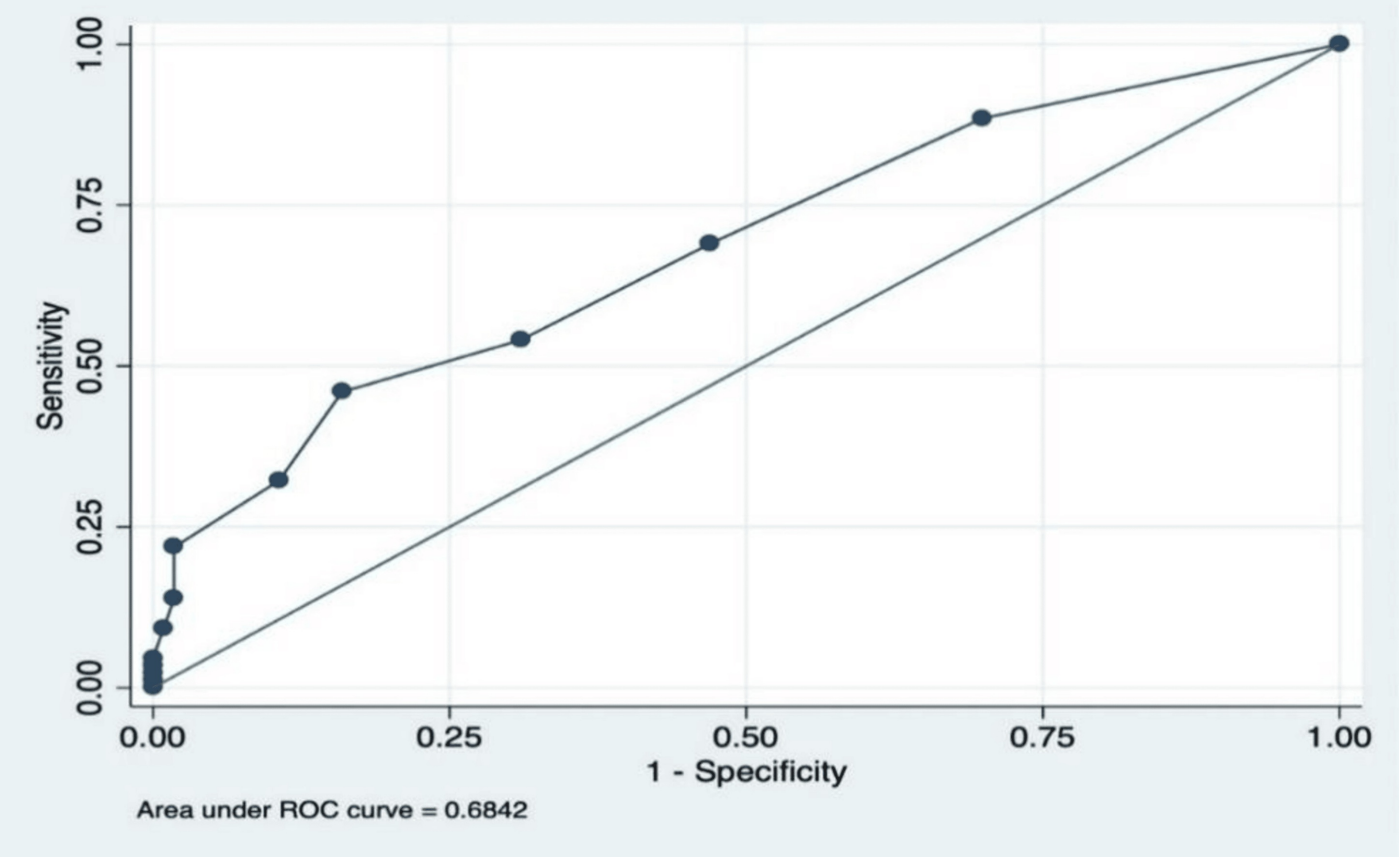
The AUC for mREMS, which is slightly higher at 0.6842, with a confidence interval from 0.61 to 0.75, indicating that mREMS offers a comparable predictive value to REMS. AUC: area under the curve; REMS: Rapid Emergency Medicine Score; mREMS: modified Rapid Emergency Medicine Score

**Figure 3:**
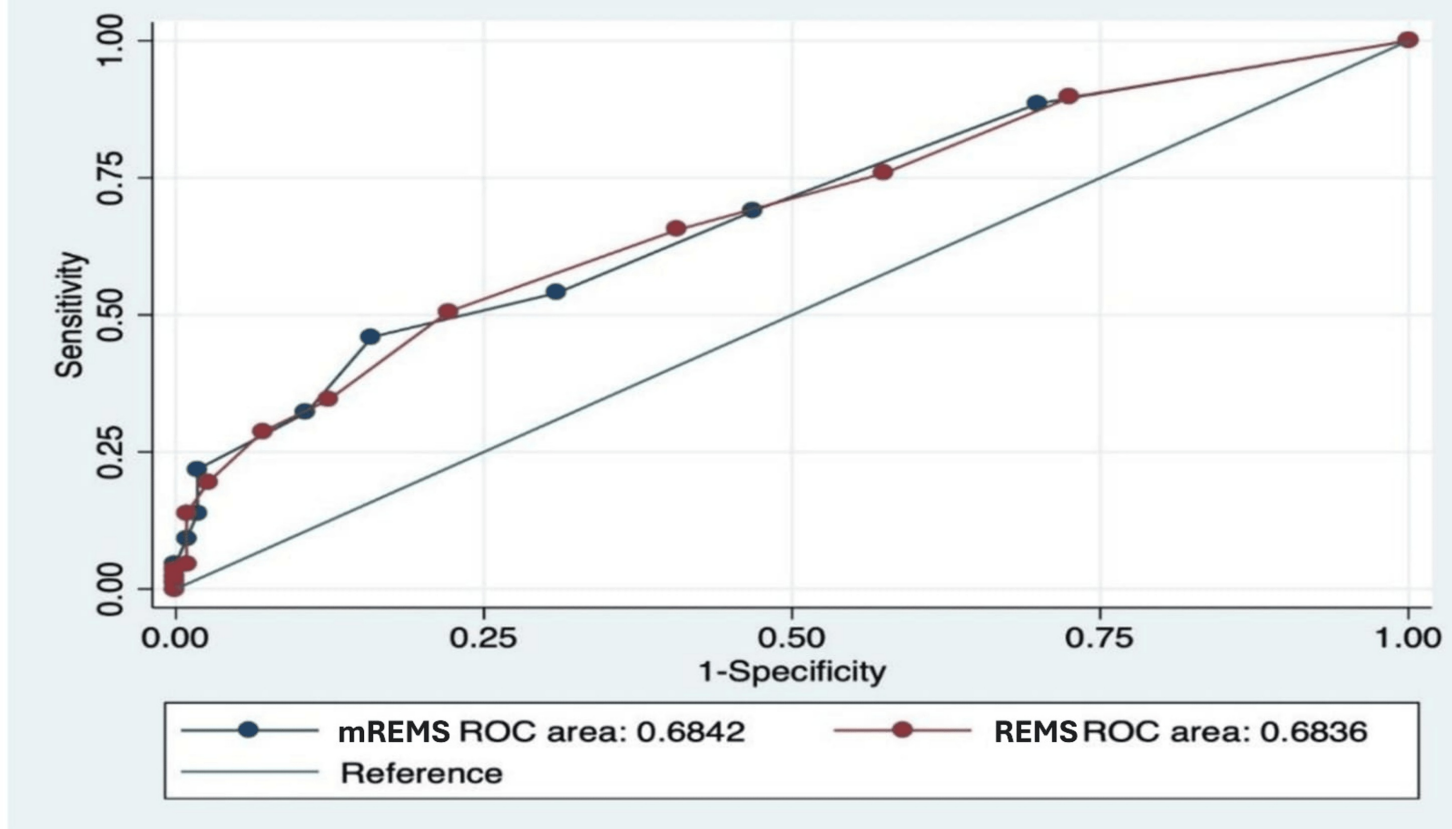
The ROC curves of REMS and mREMS visually demonstrating their similar performance in predicting the likelihood of patient admission. ROC: receiver operating characteristic; REMS: Rapid Emergency Medicine Score; mREMS: modified Rapid Emergency Medicine Score

Similarly, the mREMS was also evaluated, and its AUC was found to be 0.68 (Figure 2), with a CI spanning from 0.61 to 0.75. This suggests that the mREMS score performs at a level comparable to the original REMS score in predicting patient admission likelihood. Figure 3 compares the ROC curves of REMS and mREMS, clearly showing that both have similar performance in predicting the probability of patient admission.

The ROC curves of REMS and mREMS showed comparable performance, with both having an AUC of 0.68, indicating a moderate ability to predict patient admissions (Figure 3). The visual comparison of the curves confirms their similar predictive accuracy, suggesting that mREMS performs equivalently to REMS in assessing the likelihood of ICU admission.

## Discussion

Out of 200 patients, the proportion of male subjects was higher at 60% compared to their female counterparts. This finding was consistent with the previous literature, although the data were limited [13]. The cultural, social, and economic factors in India play a crucial role in the availability and accessibility of healthcare, which significantly impacts its success [14]. Practical conclusions, therefore, are difficult to draw. The most common comorbidities in an elderly patient are hypertension, DM, chronic obstructive pulmonary disease (COPD), cardiac failure, cancer, and cognitive impairment. These are always associated with an increase in mortality and morbidity [15]. The mean age was 72.12, with a standard deviation of 6.14. The majority, 60%, were male, echoing the established data [16].

In this study, the distribution of patients with DM was similar to that of patients with hypertension and CVA. Both crude and adjusted odds ratios indicated that these comorbidities did not affect the predictive power of the REMS score. Patients aged 65 and older were assigned a REMS score of 5, and those aged 74 and older received a score of 6, with a score of 8 or higher used as the cutoff for ICU admission. The mean age was 72.12±6.14, consistent with prior studies. Most patients had a REMS score of 0 for pulse rate, MAP, and respiratory rate, 84% had a GCS of 14-15, and 72.5% had a saturation level of greater than 89%, indicating stable clinical indicators. Future research should explore the impact of multimorbidity on mortality and morbidity.

This study found that REMS could be an effective tool for the early risk stratification of elderly patients presenting to the ED, particularly in predicting ICU admissions, morbidity, and mortality. However, the moderate sensitivity (50.57%) and moderate specificity (77.88%) of REMS suggest that it is useful but may not be sufficient when used as the sole determinant for clinical decision-making. Similar findings were observed in the study by Dundar et al. [13], which reported that REMS had a sensitivity of 70% and a specificity of 70% in predicting the need for ICU admission in elderly patients.

The CIs provide insight into the precision and reliability of the REMS score as a predictive tool for ICU admission in elderly patients. The sensitivity of the REMS score was 50.57%, with a 95% CI ranging from 43.65% to 57.5%. This indicates that while the score correctly identified just over half of the patients who required ICU admission, there is some variability in this estimate, suggesting only moderate reliability in detecting true positives. The specificity was 77.88% (95% CI: 72.12-83.63%), indicating a relatively higher accuracy in identifying patients who did not require ICU care. The narrow range of the CI for specificity suggests greater consistency in this measure. The positive predictive value was 63.77%, with a CI of 57.11-70.43%, indicating a moderate likelihood that patients with a high REMS score truly required ICU admission. Similarly, the negative predictive value was 67.18% (95% CI: 60.67-73.68%), suggesting that approximately two-thirds of the patients with lower REMS scores did not progress to ICU-level care.

Although our study's sensitivity was lower, this could be attributed to the different patient demographics and possibly the smaller sample size, as this study involved only 200 patients compared to the larger cohorts seen in prior studies. Despite the lower sensitivity in our study, REMS demonstrated a moderate ability to stratify patients into higher-risk categories, similar to findings in other studies, such as Ha et al., which showed that REMS was a reliable tool in predicting 30-day mortality with an AUC of 0.712, a significant result [17].

Regarding mortality, seven patients (3.5% of the total cohort) died during the study. This finding holds clinical significance, as all these patients had REMS scores above 8 at presentation. This outcome supports the score's ability to flag high-risk patients early. Although the overall mortality rate was relatively low, the association between elevated REMS scores and poor outcomes further justifies its use as a triage tool for identifying patients at greater risk of deterioration.

However, our study also highlights that REMS, while effective, should not be relied upon in isolation, particularly for patients with lower REMS scores. The 50% sensitivity and the 63.77% positive predictive value suggest that REMS is not entirely accurate in all scenarios. For example, 68% of patients initially admitted to the ward with a REMS score above 8 were later transferred to the ICU, suggesting that REMS may serve as an early warning signal. However, other clinical factors should be taken into consideration when making the final decision. This observation is supported by Ha et al., who noted that although REMS can predict mortality and morbidity, it is essential to incorporate clinical judgment when determining the need for intensive care in elderly patients [17].

Further reinforcing the importance of multidimensional risk assessment, our study demonstrated that common comorbidities, such as hypertension, CVA, and DM, did not significantly affect the predictive power of REMS. Dundar et al. similarly found that comorbidities in elderly patients had a minimal impact on the sensitivity and specificity of REMS for ICU admissions [13]. The consistent pattern observed across multiple studies including Ha et al. and Ghaffarzad et al. points to REMS being a robust scoring system that can accommodate diverse patient backgrounds but still requires complementary clinical tools for a comprehensive risk assessment [17,18].

Interestingly, our study's AUC for both REMS and mREMS was 0.68, which is comparable to that reported by Ha et al. [17] (AUC: 0.712) and Chatchumni et al. [19] (AUC: 0.886), but lower than that reported by Ghaffarzad et al. [18] (AUC: 0.79). While REMS and mREMS showed similar predictive abilities (AUC: 0.68), neither demonstrated strong standalone performance in our study population. These tools are valuable in providing initial risk stratification, especially in resource-limited settings, but their utility improves when combined with clinical assessment and other scoring systems. These differences in AUC values can likely be attributed to variations in study populations (e.g., specific disease groups, such as sepsis, or general ED admissions), sample sizes, and the severity of underlying conditions. It is worth noting that mREMS performed similarly to REMS in our study, reaffirming that the tool maintains its predictive power regardless of slight adjustments to scoring components, as shown in earlier work by Dundar et al. [13].

Regarding clinical implications, the REMS score can be instrumental in resource-constrained settings, such as India, where timely ICU decisions and early family counseling are critical. The study by Chatchumni et al. emphasized that scoring systems like REMS are invaluable in facilitating rapid decision-making in critical care scenarios, particularly when resources are limited, and family counseling is essential for providing realistic prognostic information [19]. The ability to accurately predict ICU admissions using a scoring system like REMS optimizes resource allocation and enables healthcare providers to engage patients' families early in the decision-making process.

Limitations

The study includes only 200 patients, which is a relatively small sample size. A larger sample size could improve statistical power and the generalizability of the findings. The study was conducted in a single tertiary care hospital, which may limit its external validity. Socioeconomic factors, access to healthcare, and quality of pre-hospital care are not accounted for, which may influence patient outcomes and limit the study's applicability across different populations.

## Conclusions

This study highlights the effectiveness of the REMS in predicting morbidity and in-hospital mortality in geriatric patients in the ED. It is particularly valuable for identifying patients at higher risk of needing ICU admission, although its moderate sensitivity (50.57%) suggests it may not catch all patients requiring intensive care. Therefore, clinicians should use REMS alongside other clinical assessments, such as comorbidities, cognitive status, and social support, to ensure a more comprehensive evaluation.

In settings with limited ICU resources, REMS can help prioritize patients who may deteriorate quickly and guide early discussions with families about possible ICU admission. However, its limited predictive value for general ward admissions and mortality indicates the need for additional factors, like frailty and functional status, to refine risk prediction further.

To the best of our knowledge, this research is the first to evaluate REMS in the geriatric ED cohort in India. The findings can inform future clinical practice by enhancing family counseling, optimizing resource allocation, and guiding clinical decisions, especially in low-resource settings. Further studies with larger, more diverse cohorts and broader inclusion criteria are needed to validate these results and explore REMS's broader applicability in managing older patients in the emergency setting.

## Appendices

Case record proforma​

​Name:

Age:

Sex:

MRD/IP number:

Serial number:

Arrival date and time:

HR (on arrival):

RR (on arrival):

BP (on arrival):

SpO2@RA (on arrival):

GCS (on arrival):​

Comorbidities: HTN/DM/CVA/Dementia

Was the patient treated outside the hospital? Yes/No

Patient disposition (from the ED):​

Admission: Ward/ITU/ICU ​

Discharge: ​

Discharge against medical advice:

Death: ​

Final diagnosis (at the time of disposition from the ED):​

Patient follow-up after 28 days of admission: ​
